# Recruitment to trials - why is it hard and how might we make it less so?

**DOI:** 10.1186/1745-6215-12-S1-A110

**Published:** 2011-12-13

**Authors:** Shaun Treweek

**Affiliations:** 1 Population Health Science, University of Dundee, Kirsty Semple Way, Dundee, DD2 4BF, UK

## Background

Recruiting participants to trials can be extremely difficult. Estimates of just how difficult vary but around half of trials fail to recruit their original target sample size. Poor recruitment can lead to an underpowered study, which may report clinically relevant effects to be statistically non-significant. A non-significant finding increases the risk that an effective intervention will be abandoned before its true value is established, or that there will be a delay in demonstrating this value while more trials or meta-analyses are done.

## Why is recruitment a problem?

Reviews of barriers and enablers to recruitment do exist, as do case studies linked to individual trials. Trial procedures that are time consuming for both clinicians and patient will adversely affect recruitment, as will trial protocols that are a long way from current routines. Not knowing how many people are likely to be eligible for the trial before starting it will lead to disappointment known as Lasagna’s Law: potential participants vanish as soon as the trial starts.

## What recruitment strategies have been used already?

Systematic reviews have found promising strategies for increasing recruitment to trials: telephone reminders; requiring potential participants to opt-out of being contacted by the trial team regarding taking part in a trial, rather than them having to opt-in, and open designs. However, recruitment strategies are rarely linked explicitly to context-specific barriers and enablers to recruitment, something that could reasonably be expected to lead to a more reproducible basis for strategy selection. Many strategies remain inadequately evaluated.

## What might help?

Trialists and methodologists should work together to link recruitment barriers and enablers to recruitment strategies. Trialists should include evaluations of their recruitment strategies in their trials and funders should support this because the number of interventions that have been rigorously evaluated in the context of a real trial is low. Creating registers of individuals interested in research participation would ease recruitment problems for many trials, especially if linked to other clinical databases to support inclusion criteria checks and recruitment planning. Methodological work around thinking of a trial as a product that needs to be marketed to participants, rather than assuming the value of the trial is obvious to all, is worthy of further investigation.

## Conclusions

Trialists and methodologists can help themselves and future recipients of healthcare innovations by making better use of existing research evidence on recruitment and by explicitly aiming to plug gaps in this evidence where they exist.

